# Could Perinatal Asphyxia Induce a Synaptopathy? New Highlights from an Experimental Model

**DOI:** 10.1155/2017/3436943

**Published:** 2017-02-23

**Authors:** María Inés Herrera, Matilde Otero-Losada, Lucas Daniel Udovin, Carlos Kusnier, Rodolfo Kölliker-Frers, Wanderley de Souza, Francisco Capani

**Affiliations:** ^1^Centro de Investigaciones en Psicología y Psicopedagogía, Facultad de Psicología, Universidad Católica Argentina, Buenos Aires, Argentina; ^2^Instituto de Investigaciones Cardiológicas (ININCA), UBA-CONICET, CABA, Buenos Aires, Argentina; ^3^Laboratório de Ultraestrutura Celular Hertha Meyer, Instituto de Biofísica Carlos Chagas Filho, Universidade Federal do Rio de Janeiro (UFRJ), Rio de Janeiro, RJ, Brazil; ^4^Departamento de Biología, Universidad Argentina JF Kennedy, Buenos Aires, Argentina; ^5^Universidad Autónoma de Chile, Santiago de Chile, Chile

## Abstract

Birth asphyxia also termed perinatal asphyxia is an obstetric complication that strongly affects brain structure and function. Central nervous system is highly susceptible to oxidative damage caused by perinatal asphyxia while activation and maturity of the proper pathways are relevant to avoiding abnormal neural development. Perinatal asphyxia is associated with high morbimortality in term and preterm neonates. Although several studies have demonstrated a variety of biochemical and molecular pathways involved in perinatal asphyxia physiopathology, little is known about the synaptic alterations induced by perinatal asphyxia. Nearly 25% of the newborns who survive perinatal asphyxia develop neurological disorders such as cerebral palsy and certain neurodevelopmental and learning disabilities where synaptic connectivity disturbances may be involved. Accordingly, here we review and discuss the association of possible synaptic dysfunction with perinatal asphyxia on the basis of updated evidence from an experimental model.

## 1. Introduction

The fascinating architectural organization of the brain is characterized by its exquisite anatomical and functional details and hierarchical network levels [1]. Synapses are highly specialized structures that allow electrochemical signals to convey tidily packaged information through the nervous system. Consequently, information from both the external and internal environment can be detected and processed and behaviour adjusted including body functions, memories, and emotions [2]. Thus, flawless synapses wiring and connectivity patterns must be preserved in order to keep messages communication unaltered and subside healthy neurophysiology. Actually, minimal disturbances of function are eventually expressed as brain disorders. By now, increasing evidence demonstrates the relevance of synapse dysfunction as a major determinant of many neurological diseases. Following this concept hereof, synaptopathies are by now treated as brain diseases themselves characterized by shared prevailing etiopathology [2]. Unfortunately, nervous cells do not divide neither are they replaced after they die off or get damaged except for a few cases. By the same token, from a prognostic viewpoint, synaptopathies are likely irreversible [1].

Synaptic networks enable organizing packaged and properly arranged information into, within, and out from the brain. Temporal spatial appearance, maturation, and specialization of synaptic units have played a key role in not only biological evolution across species, but the possibility of high cognitive and refined locomotor processes in vertebrates as well [3]. On the one hand, modulation of synapse activity constitutes a major strategy to maintain brain homeostasis. On the other hand, slight though persistent disturbances in synapse physiology are likely to underlie major defects which are eventually expressed as brain disorders. The increasingly used term “synaptopathy” refers to brain disorders underlying synaptic dysfunction. The term dates back to a review by the Brundin Laboratory discussing Huntington's disease as a result of synaptic failure [4]. In this review authors suggested that distorted synaptic communication could bring on the early symptoms of Huntington's disease and trigger neuronal death in later stages of the illness [4]. In its broadest sense the term synaptopathy refers to any perturbation in which aberrant mechanisms correlate with synaptic dysfunction regardless of its pathophysiological origin. This may result in a baffled use of the term “synaptopathy” masking whether synaptic dysfunction is the cause or consequence of a particular pathophysiological sequence of events.

Synaptic dysfunction has been consistently documented as a leading determinant in several neurodegenerative diseases and neurodevelopmental disorders (NDDs) (e.g., autism spectrum disorders, Down's syndrome, startle disease, and epilepsy) [5]. The time interval around birth is usually considered the elapsed time between 5 months before and 1 month after delivery or perinatal period in man; it is critical and presents with extreme sensitivity to disturbing factors. Accordingly, the emergence of risk factors at this time may affect the normal synaptic network structure and function which may be symptomatically expressed later in life provided epigenetic environment is presented [6]. Perinatal asphyxia (PA) is an obstetric complication derived from impaired gas exchange [7] resulting in progressive hypercapnia and hypoxemia if unattended. Perinatal asphyxia inevitably leads to abnormal brain development and neurological morbidity [6–12]. Certainly, robust literature has been produced showing evidence of the etiopathogenic role of PA in epilepsy [13], cerebral palsy [14–17], mental retardation [18], attention deficit disorder [19, 20], and schizophrenia [21, 22].

Here we present a critical overview of synapse dysfunction associated with PA illustrated with key findings obtained using an experimental model. We discuss possible causes for synapse failure as putative common denominators. Comprehension of the molecular underpinnings leading to synaptic dysfunction will aid in the development of tailored synapse-targeted therapies for neurological disorders.

## 2. Experimental Model of Perinatal Asphyxia: Early Damage in CNS

**Figure 1:**
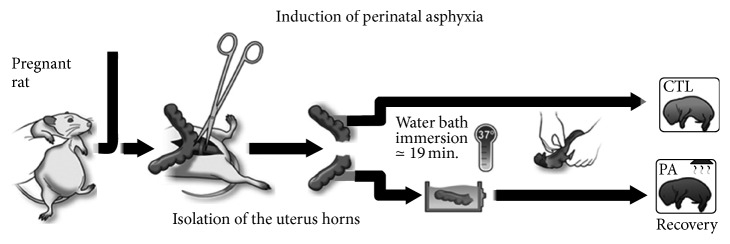
Illustration showing the main steps involved in the induction of PA. Adapted from [23].

In 1991 a global model of PA was developed at the Karolinska Institutet, Sweden. It represented a novel method to produce general asphyxia using rats which was not available by then. It consisted in performing a delayed caesarean section on pregnant rats resembling a delivery labour causing asphyctic lesions in newborns. Asphyxia was induced by immersing fetus-containing uterus horns in a water bath at 37 degrees C for 14-15 min, 15-16 min, and 16-17 min [30]. Ever since, several laboratories have used this established model of PA that induces modifications in the central nervous system (CNS) which are characterized by cortical, hippocampal, and striatal loss of neurons along with behavioural deterioration as well [30–32]. When the water bath lasts about 19 min, mortality rate increases (±40%) (Figure 1) [30, 31].

As Bjelke et al. suggested, this model is highly reproducible and easily performed. It resembles normal labour minimizing the influence of surgery, anesthesia, or drugs [30]. The main advantages of this model can be summarized as follows: (a) asphyxia takes place at the time of the delivery much the same as when umbilical cord circulation is disrupted; (b) the procedure results in systemic acidosis, hypercapnia, and hypoxia as observed in global asphyxia; (c) it is not invasive avoiding the confounding interferences of surgical procedures; (d) it induces global asphyxia which affects the whole brain (cerebral hemispheres and deep structures) making the model suitable for behavioural studies [26, 33, 34].

The immature brain is highly liable to threatening environments. Since PA is induced in newborn rats its overflowing plasticity can be certainly overwhelmed [35]. During neuronal development thin dendritic ectoplasmic pseudopods or filopodia are programmed to shield actually stable dendritic spines [36] and are determinant in the establishment of neural circuits in time [37]. Not long ago, the overexpression of M6a was reported not only to induce both neurites formation and increase in filopodium/spine density in primary cultures of rat hippocampus [38] but also to be involved in synaptogenesis [39]. Another report showed that activation by the phosphoinositide 3-kinase (a serine/threonine kinase) leading to glycogen synthase kinase 3 activation through the PI3K/Akt/GSK3 pathway was required in these processes [40]. Regulatory alterations were found in hippocampal dendritic spines cytoskeleton as prevailing features associated with memory disorders [41] whereby there is fair agreement that the hippocampus might be involved in the pathophysiology of NDDs as proposed by different studies [42, 43]. Abnormal neural network formation may be the consequence of atypical neurogenesis [44]. Many studies have focused on the analysis and discussion of cell survival and death mechanisms [45]. Nevertheless, our knowledge on the precise events triggered by PA that lead to NDDs in due time is insufficient.

Neuronal plasticity is derived from the intrinsic shapeable quality of brain tissue and it comprises neurogenesis and programmed cell death along with activity-dependent synaptic plasticity. The very well-known long-term potentiation (LTP) and long-term depression (LTD) phenomena convey the plastic response to repetitive synaptic stimulation and are associated with architectural adjustment in dendritic spines and hence in neuronal circuits [46]. Overplasticity in the developing brain could lead to disability disarranging otherwise properly organized connections and rendering maladaptive neuronal circuits [47]. Perinatal asphyxia during labour and/or delivery is doomed to cause long-term disability and embodies a worrying complication in neonatal and paediatric care [48]. In view of the not so low prevalence of this complication, several studies have reported sound information on the subject using the aforementioned experimental model, which reproduces the pathophysiological processes of PA at the time of delivery [49].

## 3. Effects of Experimental Perinatal Asphyxia on Synaptic Organization

First studies using the above-mentioned model reported time-dependent loss of pyramidal cells in CA1 and CA3 brain regions. This finding evidenced that PA could affect hippocampal microarchitecture foundations and induce subsequent memory deficits. The same model of PA also led to imbalance in the dopaminergic system involved in motor behaviour and in other neurotransmission circuits as well. The results reported by Bjelke et al. in 1991 might provide an acceptable experimental approach to induce minimal brain disorder, namely, neurodevelopmental disabilities like hyperkinesia and attention deficit as usually found in children following perinatal asphyxia [30].

Several laboratories have used the Swedish experimental model. Researchers from the Netherlands, Canada, and Germany found increased density of cortical and striatal presynaptic buttons in twenty-two-month-old rats exposed to PA. Van de Berg et al. proposed that such area-specific modifications were intended to counterbalance PA-induced neuronal loss. Quite the contrary, higher presynaptic density of buttons was related to aggravation of cognitive impairment according to age [32]. The postsynaptic locus received attention too. Argentine researchers found that asphyctic rats developed an increase in striatal synapses disarrangement and thickening in postsynaptic densities (PSDs). Perinatal asphyxia also compromised hippocampal PSDs according to severity and duration of the hypoxic insult. Lowering the temperature of the experimental setting was reported to effectively prevent synaptic changes [50]. This group had previously observed that PA induced cytoarchitectural changes in the corpus striatum and that detrimental changes could be prevented by hypothermia as well [51].

In our laboratory we found long-term misfolding and aggregation of proteins in striatal PSDs of 6-month-old rats which were subjected to severe PA. Based on our findings that PSDs were highly modified and ubiquitinated, we suggested that these changes might constitute an aberrant morphological mechanism underlying synaptic dysfunction in response to PA [24]. Following our later observations, early misfolding and aggregation of striatal synaptic proteins might actually represent the triggering point of long-term neurodegenerative events. One month after induction of PA not only did we find an increment in PSDs but also it was also concomitant with high ubiquitination levels. The magnitude of the increase in PSDs was dependent on the duration and severity of the hypoxic insult [25]. In this way we were able to confirm that protein ubiquitination could serve as a useful marker for the degree of alteration of PSDs and neuronal damage as found in experimental PA [24, 25].

**Figure 2:**
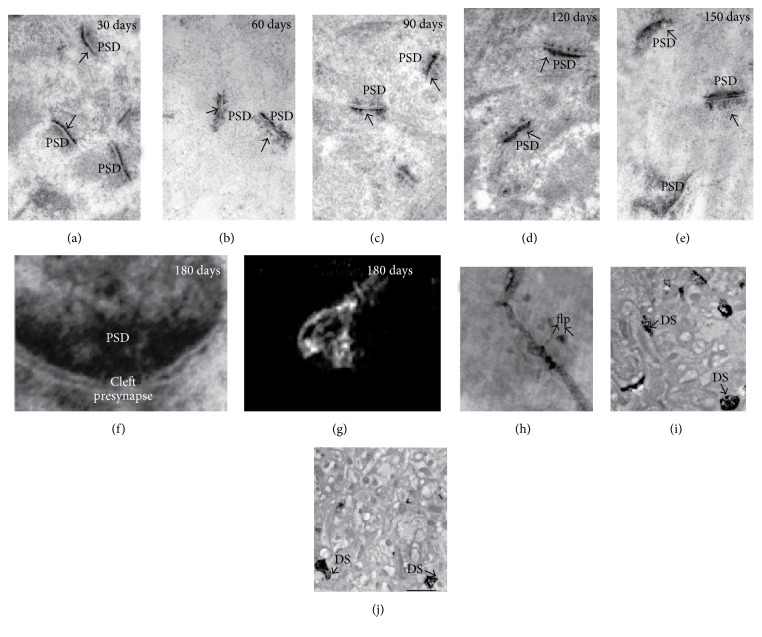
Microphotographs of synaptic terminals of the hippocampus CA1 area from animals subjected to PA. (a–e) Hippocampal synapses stained with E-PTA showing increased thickness in PSDs. (f) More consistent changes were observed in PSDs after 180 days of PA. Three-dimensional reconstruction of the asphyctic postsynaptic domain (g) showed clear signs of degeneration. (h) Dendritic shaft injected with Golgi silver staining showed filopodium after 30 days of PA (arrows). (i-j) DS stained with phalloidin eosin and photoconverted. A higher number of DS are observed at 30 days compared with 180 days after PA. PSDs = postsynaptic densities; E-PTA = ethanolic phosphotungstic acid; DS = dendritic spines. Scale bar 0.5 *μ*m.

Early synaptic alterations could be associated with striatal cytoskeletal changes induced by PA. Accumulation of F-actin was observed in dendritic spines of 1-month-old asphyctic rats. It was correlated with an increment in *β*-actin in PSDs and the number of mushroom-shaped spines and with a reduction in MAP-2 immunohistochemical labelling and the number of neurons. Therefore F-actin accumulation might represent a key cellular mechanism underlying neuronal death [26]. These striatal cytoskeletal changes were replicated 2 months after PA and could be blocked by hypothermia, which has proved to be an important therapeutic clinical tool for the outcome of PA [27]. Moreover actin modifications were observed in hippocampal cytoskeleton 4 months after induction of PA together with an increment in PSDs and the extent of ubiquitination. This finding suggested that cytoskeletal actin might play a role in PSDs alterations and ubiquitination. In addition, the number of hippocampal dendritic spines which were positive for F-actin stain decreased 4 months after PA [28] and increased 1 month after PA along with enhanced filopodium formation and synaptogenesis [29]. These findings put forward the possibility that such early attempt to rescue neural tissue via synaptogenesis and F-actin augmentation might not be actually effective. Certainly if this is so, it might lead to the late readaptive reduction in F-actin levels. These early new synapses might not be functional. Thus overplasticity might affect the proper establishment of neural circuits causing cognitive deficits as observed in behavioural tests [29].  Figure 2 shows some of these data as observed in hippocampus.  Table 1 summarizes our results in this subject.

An Austrian group was also interested in protein derangements during postnatal development following PA and attempted to identify differentially expressed proteins that might represent potential biomarkers and pharmacological targets [52]. They decided to study hippocampal proteins following their own report of postnatal changes during brain development [53]. Hippocampal protein levels were determined by a gel-based proteomic method. Results revealed an impairment of brain protein machinery as a consequence of PA. Several protein levels were altered at specific time points after the insult. Interestingly synapsin IIb levels were increased. Weitzdörfer et al. interpreted this finding as a result of both altered neurotransmitter release and compensatory synaptogenesis following PA [52]. The Swedish experimental model was selected for this study for its suitability to evaluate morphology, metabolism, neurochemistry, and long-term effects of PA [54].

A Chilean group referred to their pragmatic approach when choosing the Swedish experimental model because it induced asphyxia at the time of delivery and mimicked relevant features of the process [55]. In fact, PA is frequently associated with problematic or long-lasting delivery and the aforementioned experimental model reproduces a delayed caesarean section [30]. Furthermore this model also allows performing in vitro studies as an approach to assess particular issues elicited by the insult, such as the interference with normal neuronal network development [56].

The Chilean laboratory investigated hippocampal plasticity after PA by measuring postnatal apoptosis and neurogenesis. They suggested that PA induces short- and long-term plastic changes which are regionally specific perhaps providing a framework for functional recovery of the hippocampus. These plastic changes include delayed cell death and neurogenesis involving mitogenic proteins and also pro- and antiapoptotic proteins [57]. Recently, they investigated cell proliferation and neurogenesis as potential compensatory mechanisms for delayed cell death associated with PA using hippocampus and subventricular zone (SVZ) organotypic cultures in vitro. According to their results neurogenesis appears to be mediated by dopamine receptors and PA could hinder this mechanism in certain areas. They hypothesized that PA might cause delayed cell death by disrupting postnatal plasticity rather than triggering a neurotoxic cascade. In other words PA might modulate postnatal neurogenesis. Therefore dopamine agonists might have neuroprotective potential via facilitation of postnatal neurogenesis and restoration of damaged circuitries [58]. Certainly, in vitro studies revealed a selective decrease in the number, neurite length, and branching of dopamine neurons as a consequence of PA [59]. Likewise a reduction in neurite length and branching was observed in the hippocampus 1 month after PA along with reduced expression of pre- and postsynaptic markers (resp., synaptophysin and postsynaptic density protein 95, PSD95) [60]. Following this evidence the Chilean group has apparently confirmed and extended Bjelke et al.'s original findings about PA-induced alteration of dopamine circuitries and hippocampal architecture. In fact, the leader of the Chilean group Herrera-Marschwitz had previously worked with Bjelke and Andersson at the Karolinska Institutet, where they published several papers about the effects of PA on rat brain and the dopamine system in particular.

Researchers from the Netherlands were also interested in the effect of PA on postnatal plasticity during the first weeks of life. They used the Swedish experimental model in order to study the ontogeny of neurotrophic factors involved in the regulation of developmental plastic changes [61]. They had previously reported a delayed increase in cellular proliferation in the hippocampus 5 days after PA, likely related to an increase in neurotrophic factors caused by the injury [62]. In contrast to that observed in the adult injured brain PA induced opposing changes in nerve growth factor (NGF) and brain derived neurotrophic factor (BDNF) content in a spatiotemporal-dependent fashion. On these bases, further studies should attempt to elucidate the mechanism of action of neurotrophic factors in the developing brain after PA [61].

Most studies using the Swedish experimental model of PA have focused on grey matter pathology. However as myelin interferes with nerve conduction velocity and may affect synaptic transmission, studies on the effects of PA on myelination are relevant. The aforementioned Austrian researchers reported long-term myelination deficits in hippocampus and cerebellum of rats subjected to PA. These deficits were regularly accompanied by neuronal loss which was measured by a decrease in neurofilament immunoreactivity. This finding suggests that grey matter damage is linked to myelination deficits which should also be considered when studying mechanisms involved in the pathogenesis of NDDs following PA [63].

## 4. Conclusion

Our aim was to shed some light on the synaptic dysfunction linking PA with NDDs. Perinatal asphyxia occurs when the brain is still immature and the insult may affect initial plasticity required for the establishment of circuitries and synapses. The consumption of extra energy for the reestablishment of homeostasis might compete with the demands required for circuitries and synapsis consolidation [55]. Several studies using the Swedish experimental model have reported distinct alterations induced by PA such as thickening of both pre- [32] and postsynaptic densities [24–29, 50]; protein misfolding, aggregation, and ubiquitination [24–29]; interruption of postnatal neurogenesis [58]; reduction in neurite length and branching [59, 60]; myelination deficits [63]; and modifications in the levels of synapsin [52, 60] and neurotrophic factors [61]. Some researchers have suggested the existence of plastic changes in an attempt to counterbalance neuronal loss [29, 32, 52, 57]. However, compensatory mechanisms, namely, overplasticity, might not always be functional and may result in aggravation of cognitive impairment [32]. The inadequacy of neural circuits is irreparably accompanied by behavioural deficits [29].

Further studies should be designed in order to dissect molecular changes involved in synaptic alterations induced by PA. The Swedish experimental model of PA appears to be a useful tool of clinical relevance [26, 30, 33, 34, 54–56]. Also electrophysiological evidence is needed so as to clarify the relevance of new dysfunctional synapses and long-term neurodegeneration leading to synaptopathy following PA.

Hopefully this information will help to design new therapeutic tools, certainly a challenge for medical research in this field.

## Figures and Tables

**Table 1:** Summary of PA-induced changes from our laboratory.

Reference	Time after PA	Brain area	Main findings	Concluding remarks
Capani et al. 2009 [24]	6 months	Striatum	Thickening in PSDs and high ubiquitination levels related to injury duration and severity. Hypothermia prevented changes.	Long-term protein misfolding/aggregation in PSDs may drive synaptic dysfunction/neuronal damage.

Grimaldi et al. 2012 [25]	1 month	Striatum	Thickening in PSDs and high ubiquitination levels related to injury duration and severity.	Early misfolding/aggregation of synaptic proteins could induce long-term changes and neurodegeneration.

Saraceno et al. 2012 [26]	1 month	Striatum	Accumulation of cytoskeletal F-actin in dendritic spines. Increased number of mushroom-shaped spines. Reduced number of neurons.	Early synaptic alteration/neuronal damage might be linked to cytoskeletal F-actin accumulation.

Muñiz et al. 2014 [27]	2 months	Striatum	Increased number of mushroom-shaped F-actin dendritic spines. Hypothermia prevented changes.	Sustained synaptic and cytoskeletal changes were found.

Saraceno et al. 2012 [28]	4 months	Hippocampus	Thickening in PSDs and high ubiquitination levels. Reduced number of F-actin stained spines.	Long-term actin cytoskeleton might play a role in PA-induced PSD alterations.

Saraceno et al. 2016 [29]	1 month	Hippocampus	Thickening in PSDs and increased number of F-actin stained spines. Enhanced filopodium formation and synaptogenesis. Habituation memory changes.	Likely dysfunctional synapses might result in late readaptive decrease in F-actin levels. Overplasticity might affect the adequate establishment of neural circuits.

PSDs: postsynaptic densities. See text for more details.
